# Vascular access site management during electrophysiology procedures: a European Heart Rhythm Association survey

**DOI:** 10.1093/europace/euaf117

**Published:** 2025-06-16

**Authors:** Mark T Mills, Dhiraj Gupta, Vishal Luther, Maura M Zylla, Piotr Futyma, Laura Perrotta, Michal Mazurek, Christian-Hendrick Heeger, Lina Marcantoni, Andreas Metzner, Julian K R Chun

**Affiliations:** Liverpool Centre for Cardiovascular Science at University of Liverpool, Liverpool John Moores University and Liverpool Heart & Chest Hospital, William Henry Duncan Building, West Derby Street, Liverpool L7 8XL, UK; Department of Cardiology, Liverpool Heart & Chest Hospital NHS Foundation Trust, Thomas Drive, Liverpool L14 3PE, UK; Liverpool Centre for Cardiovascular Science at University of Liverpool, Liverpool John Moores University and Liverpool Heart & Chest Hospital, William Henry Duncan Building, West Derby Street, Liverpool L7 8XL, UK; Department of Cardiology, Liverpool Heart & Chest Hospital NHS Foundation Trust, Thomas Drive, Liverpool L14 3PE, UK; Liverpool Centre for Cardiovascular Science at University of Liverpool, Liverpool John Moores University and Liverpool Heart & Chest Hospital, William Henry Duncan Building, West Derby Street, Liverpool L7 8XL, UK; Department of Cardiology, Liverpool Heart & Chest Hospital NHS Foundation Trust, Thomas Drive, Liverpool L14 3PE, UK; Department of Cardiology, Heidelberg Center of Heart Rhythm Disorders, Medical University Hospital, Im Neuenheimer Feld 410, Heidelberg, Germany; Medical College, University of Rzeszów and St. Joseph’s Heart Rhythm Center, Rzeszów, Poland; Arrhythmia Unit, Department of Cardiology, Careggi University Hospital, Florence, Italy; 1st Department of Cardiology and Angiology, Silesian Center for Heart Diseases, Zabrze, Poland; Department of Rhythmology; Cardiology and Internal Medicine, Asklepios Klinik Hamburg Altona, Hamburg, Germany; Cardiology Department, Santa Maria della Misericordia Hospital, Rovigo, Italy; Department of Cardiology, University Heart and Vascular Center Hamburg, University Medical Center Hamburg-Eppendorf, Hamburg, Germany; Cardioangiologisches Centrum Bethanien, Agaplesion Markus Krankenhaus, Frankfurt am Main, Germany

**Keywords:** Electrophysiology, Catheter ablation, Vascular access, Haemostasis, Complications

## Abstract

**Aims:**

Reliable vascular access and haemostasis techniques are important to the safety of electrophysiology (EP) procedures. This European Heart Rhythm Association (EHRA) survey aimed to evaluate contemporary vascular access site management practices across international EP centres.

**Methods and results:**

A 30-question survey was disseminated via the EHRA between March and April 2025, with 401 responses from professionals across 51 countries. Most respondents were cardiology consultants/attendings (82.0%), with 57.3% performing over 150 EP procedures annually. Ultrasound guidance for vascular access was usually or always used by 71.7%, though 21.4% used it rarely or never, and only 17.3% had received formal ultrasound training. Institutional protocols for haemostasis were lacking in around half (46.8%) of centres. Suture-mediated closure was the most common method for haemostasis (60.4%), followed by manual compression (33.0%) and vascular closure devices (VCDs, 5.8%). The figure-of-eight suture with a hand-tied knot was the most frequently used suture technique (79.7%). Just over a third (36.0%) had experience with VCDs, typically reserved for high-risk cases. For procedures requiring transeptal access, 38.1% administered heparin before transeptal puncture, while protamine was rarely or never used by 62.1%. Anticoagulation was partially interrupted in 52.1% and continued uninterrupted in 41.1% of routine atrial fibrillation (AF) ablations. The median bed rest duration post-procedure ranged from 4 h (right-sided EP procedures) to 6 h (AF or left-sided EP procedures). The average quoted vascular complication risk during consent was 3% (inter-quartile range 1–5%).

**Conclusion:**

This survey highlights marked variation in vascular access site management during and following EP procedures, emphasizing the need for further clinical trials to inform best practice and guide future standardization efforts.

## Graphical Abstract

**Graphical Abstract:**
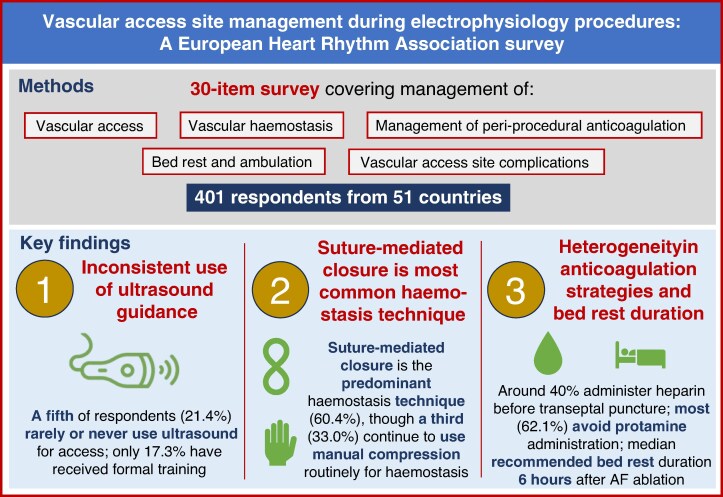

## Introduction

Over the last half century, catheter-based electrophysiology (EP) procedures have transformed the diagnosis and treatment of cardiac arrhythmias,^1^ significantly improving patient outcomes over medical therapy alone. These procedures are predominantly performed via femoral venous access, facilitating diagnostic and ablation catheter entry into the right or left cardiac chambers. Typically, one to four vascular sheaths are inserted, ranging from 5 to 17 Fr in diameter. Certain EP procedures also require arterial access, or venous access at other anatomical sites.

While technological and procedural advancements have enhanced the safety and efficacy of EP procedures, vascular access site complications remain the most frequently reported adverse events, ranging from 1 to 4% in atrial fibrillation (AF) catheter ablation.^2–9^ These range from minor issues, such as bleeding and haematoma requiring manual compression and extended bed rest, to major complications, including vascular injury necessitating transfusion, radiological or surgical intervention, or resulting in critical illness. Even in the absence of severe complications, patients may experience local discomfort, bruising, and prolonged recovery times.

Several key aspects of vascular access site management are critical to the safety of EP procedures, including techniques for vascular access,^10–12^ strategies for vascular haemostasis and closure,^13–16^ and protocols for managing periprocedural anticoagulation.^17^ Additionally, differences exist in bed rest duration and ambulation protocols following sheath removal, which may influence patient recovery and complication rates.^18,19^ Vascular access site complications remain a significant concern, reinforcing the need for optimized, evidence-based approaches to reduce risks and standardize best practices.

In this European Heart Rhythm Association (EHRA) Scientific Initiatives Committee survey, we aimed to assess current practice regarding vascular access site management during EP procedures across European centres, identifying trends, variations, and areas for potential standardization.

## Methods

### Questionnaire development and dissemination

A bespoke 30-item questionnaire was developed by the EHRA Scientific Initiatives Committee,^20^ comprising multiple-choice questions and questions requiring numerical responses. The questionnaire was reviewed, edited, and approved by all co-authors and included sections on: (i) respondents’ demographics; (ii) vascular access; (iii) vascular haemostasis and closure; (iv) management of periprocedural anticoagulation; (v) bed rest and ambulation protocols; and (vi) vascular access site complications. The full questionnaire is provided in the Supplementary material online, Appendix  *S1*.

The link to the online questionnaire was distributed to the EHRA and EHRA Young EP communities via email and also promoted via social media, between 24 March and 21 April 2025. The survey was open to all healthcare professionals undertaking EP procedures (including—but not limited to—physicians, nurses, and physician associates). Response was voluntary, anonymous, and General Data Protection Regulation compliant.

### Statistical analysis

Continuous variables were non-normally distributed and presented as median and inter-quartile range (IQR). Categorical variables are reported as counts and percentages. Missing responses to specific questions were excluded, with the resulting sample size reported. Statistical analysis was conducted in SPSS (version 29; IBM).

## Results

Of the 481 total responses received, 80 questionnaires were excluded due to lack of study-relevant information (containing no responses beyond country of the respondent and/or the number of EP procedures performed), leaving 401 questionnaires for analysis.

**Figure 1:**
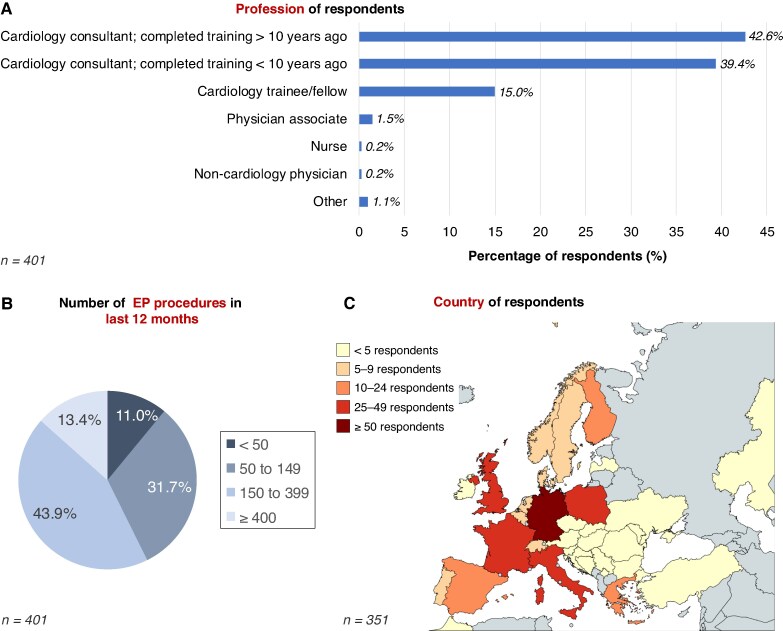
Demographics of respondents. (*A*) Professional category of respondents. (*B*) Number of EP procedures in the preceding 12 months. (*C*) Country of work of respondents (created with MapChart.net). EP, electrophysiology.

The majority of respondents were cardiology consultants or attendings, either more than 10 years post-completion of training (42.6%) or within 10 years of completion of training (39.4%), while 15.0% were cardiology trainees or fellows (*Figure 1A*). Approximately one-third (31.7%) of respondents reported performing between 50 and 149 EP procedures in the preceding 12 months, and 43.9% reported performing between 150 and 399 procedures (*Figure 1B*). Respondents represented 51 countries, with the distribution of responses per country shown in *Figure 1C*. The five countries with the highest number of respondents were Germany (16.5%), France (12.8%), the UK (9.1%), Poland (9.1%), and Italy (9.1%).

### Vascular access

**Figure 2:**
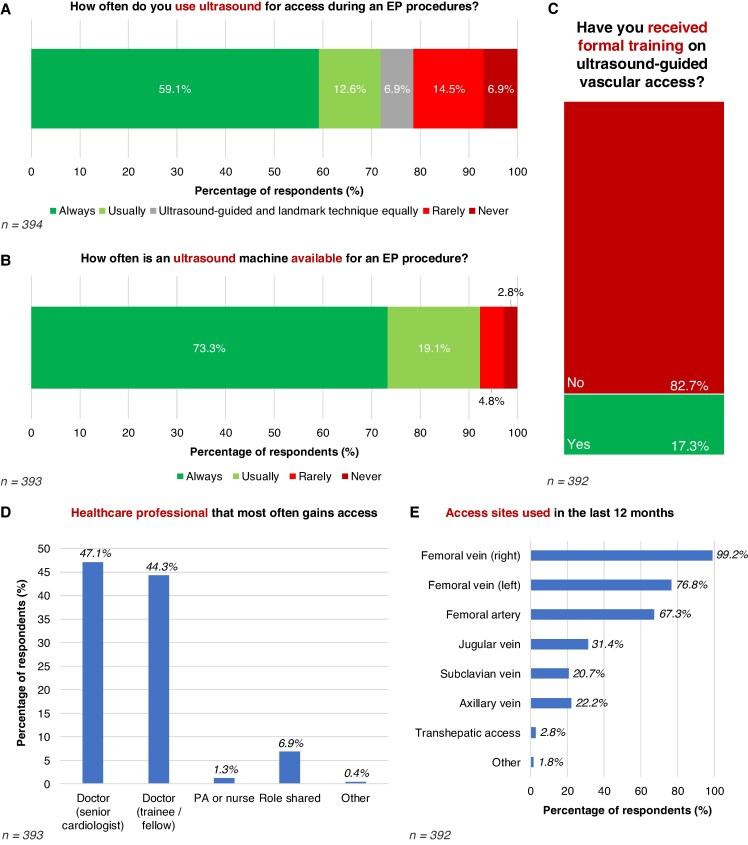
Vascular access. (*A*) Use of ultrasound. (*B*) Availability of ultrasound during an EP procedure. (*C*) Training in ultrasound-guided access. (*D*) Healthcare professional gaining vascular access. (*E*) Vascular access sites used in the preceding 12 months. EP, electrophysiology; PA: physician associate.

The majority of respondents reported always (59.1%) or usually (12.6%) using ultrasound guidance for vascular access during EP procedures, while 21.4% reported using ultrasound rarely or never (*Figure 2A*). Most respondents (73.3%) indicated that an ultrasound machine is always available when obtaining vascular access (*Figure 2B*). However, only 17.3% of respondents had received formal training in ultrasound-guided vascular access, such as attending a practical course or undergoing hands-on training with a sign-off process (*Figure 2C*).

Vascular access was most commonly performed by physicians, either a senior cardiologist (47.1%) or a trainee/fellow (44.3%) (*Figure 2D*). In the preceding 12 months, 99.2% of respondents reported using right femoral venous access, 76.8% left femoral venous access, 67.2% femoral arterial access, 31.4% jugular venous access, 22.2% axillary venous access, and 20.7% subclavian venous access (*Figure 2E*). For a routine diagnostic EP study, the most common access site was unilateral femoral vein access only (80.1%), followed by bilateral femoral vein access (16.3%), and femoral vein access combined with another access site (e.g. subclavian or jugular vein) in 3.3% of cases.

### Vascular haemostasis and closure

**Figure 3:**
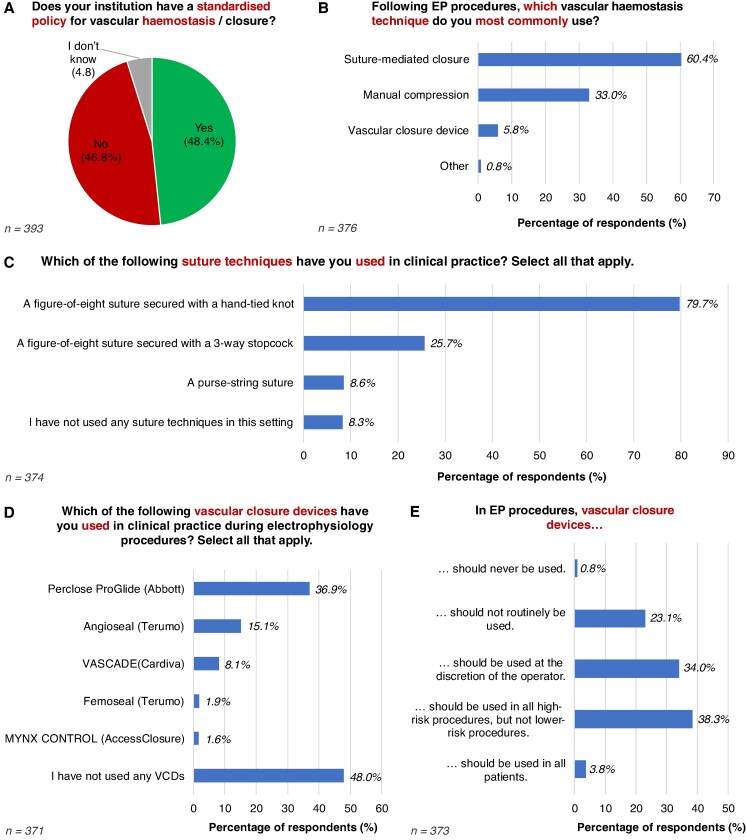
Vascular haemostasis and closure. (*A*) Existence of standardized institutional policy on vascular haemostasis or closure. (*B*) Most commonly used vascular haemostasis technique. (*C*) Use of suture-based haemostasis techniques. (*D*) Use of VCDs. (*E*) Opinion on VCDs. VCDs, vascular closure devices.

Less than half of respondents (48.4%) reported that their institution had a standardized protocol for haemostasis/vascular closure after an EP procedure (*Figure 3A*). A majority had clinical experience with suture-mediated closure (82.9%) and manual compression (79.3%), while 36.0% had experience using a vascular closure device (VCD) during EP procedures. In routine practice, the most commonly employed haemostasis method was suture-mediated closure (60.4%), followed by manual compression (33.0%) and VCDs (5.8%) (*Figure 3B*).

When manual compression is used, it was most frequently performed by a doctor, either a trainee/fellow (36.9%) or senior cardiologist (23.3%). This was followed by nurses (22.7%), physician associates (1.0%), or other healthcare professional (1.0%). In 15.1% of cases, manual compression was shared equally amongst different professional groups. For anticoagulated patients undergoing AF ablation, respondents estimated a median time of 10 min (IQR 5–15 min) to achieve haemostasis using manual compression.

Amongst suture-based techniques, the most commonly used method was the figure-of-eight (Fo8) suture secured with a hand-tied knot (79.7%), followed by the Fo8 suture with a 3-way stopcock (25.7%) and the purse-string suture (8.6%) (*Figure 3C*).

Nearly half of respondents (48.0%) reported no prior use of a VCD during an EP procedure (*Figure 3D*). The most commonly used VCD was the Perclose ProGlide (Abbott; 36.9%), followed by Angioseal (Terumo; 15.1%), VASCADE (Cardiva; 8.1%), Femoseal (Terumo; 1.9%), and Mynx Control (AccessClosure; 1.6%) (*Figure 3D*). Across all EP procedures performed in the preceding 12 months, respondents estimated using a VCD in 3% of their EP procedures (IQR 0–10%). The average estimated cost of a VCD was 263€ (IQR 175.5–500€).

Regarding the appropriate use of VCDs, 38.3% of respondents believed they should be reserved for high-risk EP procedures (e.g. large-bore or arterial access), while 34.0% felt that their use should be at the operator's discretion. A further 23.1% stated that VCDs should not be used routinely, while 3.8% believed they should be used in all patients (*Figure 3E*).

Approximately half of respondents (54.9%) always applied a pressure bandage after achieving haemostasis, while 28.7% sometimes did so, and 16.4% never applied one. When used, the median duration the pressure bandage was left in place was 6 h (IQR 4−8 h).

### Management of periprocedural anticoagulation

**Figure 4:**
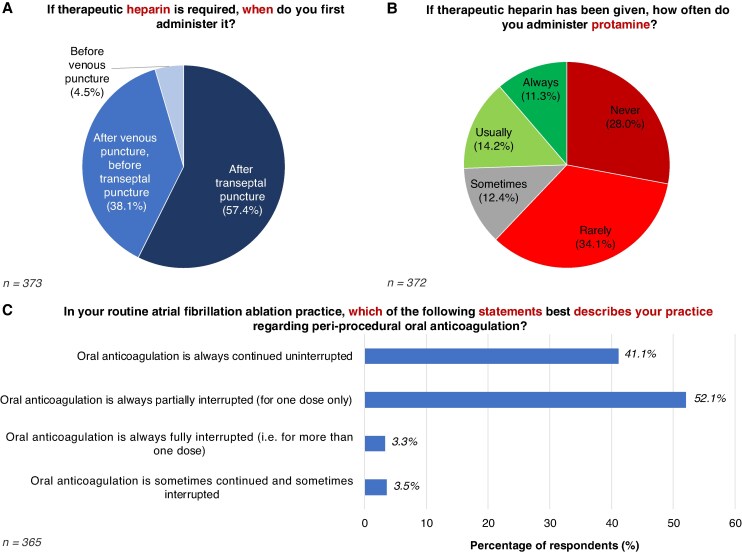
Management of periprocedural anticoagulation. (*A*) Timing of heparin administration. (*B*) Use of protamine. (*C*) Periprocedural oral anticoagulation management.

In EP procedures requiring transeptal access, 57.4% of respondents reported administering therapeutic heparin after transeptal puncture, 38.1% after venous puncture but before transeptal puncture, and 4.5% before venous puncture (*Figure 4A*).

When therapeutic dose heparin was administered intra-procedurally, most respondents rarely (34.1%) or never (28.0%) used a reversal agent such as protamine, while 14.2% usually and 11.3% always administered it (*Figure 4B*). Amongst those who used protamine, 57.9% administered a partial dose to partially reverse heparinization, 26.9% gave full dose for complete reversal, and 15.2% used either approach depending on the case.

For routine AF ablation, approximately half of respondents (52.1%) reported partially interrupting anticoagulation for one dose only, while 41.1% continued uninterrupted anticoagulation (*Figure 4C*).

### Bed rest duration prior to ambulation

Most respondents (83.8%) reported that their centre had a standardized protocol for bed rest duration following EP procedures (see Supplementary material online, Appendix  *S2*). Recommended bed rest durations for three different procedures are summarized in Supplementary material online, Appendix  *S2*: right-sided EP procedures without therapeutic anticoagulation: median 4 h, IQR 3–6 h; AF ablation: median 6 h, IQR 4–8 h; left-sided EP procedures requiring femoral arterial access: median 6 h, IQR 5–12 h.

### Vascular access site complications

**Figure 5:**
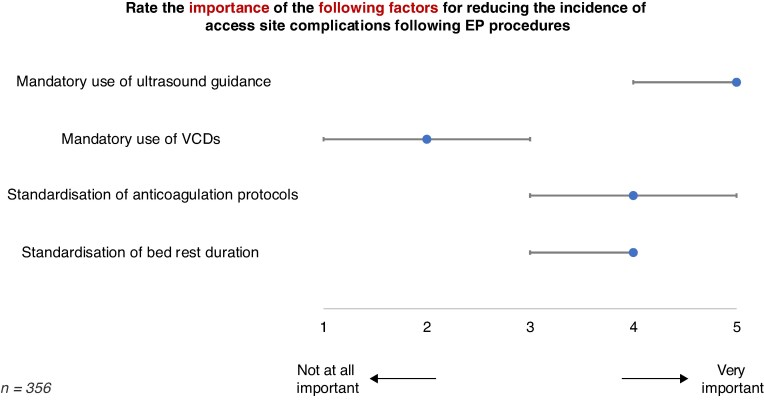
Importance of factors in reducing vascular access site complications. Median, inter-quartile range. EP, electrophysiology; VCDs, vascular closure devices.

When consenting patients prior to an EP procedure, respondents quoted the average risk of vascular complications to be 3% (IQR 1–5%). Respondents were asked to rate the importance of four strategies for reducing access site complications on a scale from 1 (not at all important) to 5 (very important). The mandatory use of ultrasound was considered the most important factor (median 5, IQR 4–5), followed by standardization of periprocedural anticoagulation protocols (median 4, IQR 3–5) and standardization of bed rest duration (median 4, IQR 3–4). Mandatory use of VCDs was identified as the least important factor (median 2, IQR 1–3) (*Figure 5*).

## Discussion

This survey provides a comprehensive assessment of vascular access site management practices during EP procedures across international centres. Despite rapid advances in technology and increasing procedural volumes, our findings reveal considerable heterogeneity in access techniques, haemostasis strategies, and post-procedural care. These results highlight important gaps in evidence and support the need for prospective clinical studies to determine best practices and inform future efforts towards standardization.

### Inconsistent use of ultrasound guidance

Ultrasound guidance is widely advocated for vascular access due to its association with reduced complications,^21^ yet a fifth of respondents (21.4%) in our survey reported rarely or never using it. This is comparable to the findings of a previous EHRA survey from 2023, in which 28% of respondents reported never using ultrasound guidance for femoral venous puncture.^22^ Multiple studies have demonstrated the benefits of ultrasound-guided venous access in EP procedures. In the ULTRA-FAST randomized controlled trial (RCT), ultrasound guidance significantly improved procedural efficiency—reducing puncture time (288 vs. 369 s, *P* < 0.001) and increasing first-pass access (74 vs. 20%, *P* < 0.001)—although it did it not significantly reduce complications, likely due to limited statistical power.^11^ However, a systematic review and meta-analysis of 4065 patients found that ultrasound guidance significantly reduced major vascular complications by 60% (relative risk 0.40; 95% confidence interval, 0.28–0.91) and minor complications by 66% (relative risk, 0.34; 95% confidence interval, 0.15–0.78) compared with anatomical landmark techniques.^10^

Furthermore, only 17.3% of respondents in our survey had received formal training in ultrasound-guided access, suggesting that this skill is predominantly acquired ‘on the job’, potentially without exposure to optimal techniques or best practices. This lack of formal training may help explain its underutilization. Given increasing procedural volumes, integrating mandatory ultrasound training into EP fellowship programmes and procedural credentialing could offer substantial patient benefit.

### Variation in haemostasis and vascular closure techniques

Our survey demonstrates a lack of standardized haemostasis protocols in nearly half (46.8%) of centres. Suture-mediated closure techniques were the most frequently used (60.4%), but manual compression remains common (33.0%). Routine use of VCDs was reported by only 5.8% of respondents, although over a third (36.0%) had experience using them.

The Fo8 suture with a hand-tied knot remains the predominant suture technique. This method has demonstrated safety and efficacy, including for large-bore venous sheaths (up to 15 Fr) used during cryoballoon ablation.^15^ Its widespread use likely reflects its simplicity, low cost, and reproducibility. Multiple observational studies have evaluated the role of a Fo8 suture for femoral haemostasis in EP procedures, particularly amongst patients undergoing AF ablation.^15,23–28^ Two small RCTs comparing the Fo8 suture to manual compression have shown improved outcomes, although both were underpowered for major complications.^29,30^ The largest observational study to date (1089 patients) found that a modified Fo8 technique—where the suture is secured with a three-way stopcock rather than hand-tied knot—was associated with the lowest rate of vascular access site complications.^28^ Notably, only a quarter (25.7%) of respondents in our survey reported experience with this modified technique, which the present authors advocate for as a simple, effective, and elegant technique.

Conversely, the use of VCDs remains relatively limited, accounting for only 3% of all EP procedures reported in our survey, despite emerging evidence supporting their utility.^14,16,31^ Notably, 38.3% of respondents felt that VCDs should be reserved for high-risk or large-bore procedures, suggesting selective rather than universal use. In the STYLE-AF RCT, the use of the Perclose™ device resulted in earlier ambulation (109 vs. 269 min, *P* < 0.001) and a trend towards fewer minor access site complications (11.1 vs. 24.2%, *P* = 0.063) compared with the Fo8 suture in patients undergoing AF ablation.^16^ Similarly, the PRO-PVI study demonstrated effective haemostasis and facilitated early same-day discharge using Perclose™ in an ambulatory workflow for pulmonary vein isolation.^14^ Further, a large propensity score-matched analysis of over 28 000 patients undergoing AF ablation found a lower incidence of vascular access site-related complications with VCD use during both early (≤7 days) and extended (8–30 days) follow-up.^28^

Several factors may explain the limited uptake of VCDs in routine practice. These include high device cost, concerns over off-label use in the venous system, and limited operator experience, reflected in our survey findings. Further RCTs and cost-effectiveness analyses are needed to better define the optimal role of VCDs in EP procedures.

### Heterogeneity in anticoagulation strategies

Management of periprocedural anticoagulation in EP procedures remains variable. Our survey reveals significant heterogeneity in the timing of heparin administration, use of reversal agents, and interruption of oral anticoagulants.

Around 40% of respondents administered heparin prior to transseptal puncture, a strategy supported by evidence linking early anticoagulation to reduced thromboembolic risk.^32^ For instance, a study of over 500 patients undergoing AF ablation demonstrated that initiating heparin before the first transseptal puncture significantly decreased the incidence of intracardiac thrombus detected by intracardiac echocardiography (from 9.1% when heparin was administered after two transeptal punctures to 0% when given beforehand).^32^ Despite this, 57.4% of survey respondents delayed heparin until after transseptal puncture, likely due to the perceived risks related to inadvertent pericardial puncture.

The adoption of uninterrupted anticoagulation strategies during AF ablation has been reinforced by trials demonstrating low stroke and bleeding risks with this approach. The RE-CIRCUIT RCT found that uninterrupted dabigatran was associated with significantly fewer major bleeding events than uninterrupted warfarin, without an increase in thromboembolic complications.^33^ In our survey, however, 41.1% of respondents continued anticoagulation uninterrupted, while 52.1% withheld a single dose.

Protamine use to reverse heparin was uncommon in our survey, with 62.1% of respondents rarely or never using it. While reversal may reduce bleeding and facilitate haemostasis, protamine itself carries risks, including hypotension, bradycardia, and, rarely, cardiac arrest,^17^ which may deter its use. A tailored approach considering sheath size, bleeding risk, and patient comorbidities may be most appropriate, though guidance remains limited.

### Complications and post-procedural bed rest

The median quoted risk of vascular access site complications during patient consent (3%) closely aligns with published rates of 1–4% reported in AF ablation studies,^2–9^ suggesting that current consent practices broadly reflect real-world data and perceived procedural risk amongst operators.

Although 83.8% of respondents reported a standardized institutional protocol, recommended post-procedural bed rest durations varied widely. Striking the right balance is important, as excessive bed rest can impair early recovery and prolong hospital stay, whereas premature ambulation may increase bleeding risk.

In the STYLE-AF and PRO-PVI studies, early ambulation (within 2–3 h) following VCD-assisted closure was safe and associated with high patient satisfaction.^14,16^ These findings challenge the traditional approach of prolonged bed rest, particularly when reliable haemostasis techniques are employed. Integrating such protocols into evidence-based consensus guidelines has the potential to promote earlier mobilization and shorten hospital stays, without compromising safety.

### Limitations

This study has several limitations. First, despite receiving over 400 responses from a broad geographic distribution, the survey is subject to selection bias. Respondents with a particular interest or expertise in vascular access site management may have been more likely to participate, potentially limiting generalizability. Second, all data were self-reported and may reflect over- or under-estimation of adherence to recommended practices (e.g. ultrasound guidance). Additionally, responses reflect individual perspectives, which may not accurately represent centre-wide protocols, particularly in larger or multi-operator institutions. Third, several questions did not differentiate between procedure types (e.g. diagnostic studies vs. complex ablations), which may influence interpretation of responses regarding haemostasis, anticoagulation, or complication risk. Moreover, the survey's cross-sectional design captures practices at a single time point, limiting assessment of temporal trends. Subgroup analyses by country or procedural volume were not performed due to variability in response rates and the risk of introducing bias. Finally, this study did not seek to evaluate clinical outcomes or compare the efficacy and safety of vascular access strategies; further prospective studies and randomized trials are needed to define optimal approaches in EP procedures.

## Conclusions

This EHRA survey highlights significant variability in vascular access site management across European EP centres. Despite the availability of proven techniques such as ultrasound guidance and Fo8 suture closure, gaps remain in training and implementation. These findings emphasize the need for further research and targeted education to address practice variation and inform future consensus on optimal strategies for improving safety and patient outcomes in modern EP practice.

## Supplementary Material

euaf117_Supplementary_Data

## Data Availability

The data underlying this article will be shared on reasonable request to the corresponding author.
