# Supplementary material for: Vascular access site management during electrophysiology procedures: a European Heart Rhythm Association survey
Source: Europace. 2025 Jun 16;27(7):euaf117. doi: 10.1093/europace/euaf117 (PMC12212052; doi:10.1093/europace/euaf117)

**Supplementary Material – Appendix 1: Full survey**

| **Vascular access site management during electrophysiology procedures – an EHRA survey** |
| --- |

**Introduction**

Dear colleague,

The European Heart Rhythm Association (EHRA) Scientific Initiatives Committee (SIC) is collecting information regarding vascular access site management during electrophysiology procedures. The survey is intended for any healthcare professional performing or assisting with electrophysiology procedures.

This survey includes questions on the practicalities of vascular access, vascular haemostasis, management of periprocedural anticoagulation, bed rest duration, and vascular access site complications.

On behalf of the EHRA SIC, we thank you for your participation, time and effort.

GDPR disclaimer:

Your participation is anonymous.

We will not disclose your identity to any third party.

We comply with the European General Data Protection Regulation (GDPR) 2016/679. Any personal data processed in connection with this survey will be treated confidentially and only used by the ESC for the purposes of market research and not for promotion. Survey results will be kept for a maximum of 48 months for analysis and quality control purposes. We take all reasonable care to prevent any unauthorised access to your personal data. We respect your privacy and your right to access, modify, or remove your personal data. At any time, you can ask to know what personal data is being held. If you have any questions about data protection or require further information, please contact our data protection officer (DPO) at [dpo@escardio.org](mailto:dpo@escardio.org).

You have the right to end your participation in this survey at any time.

**1. Please confirm that you have read the above and agree to participate in this survey.**

1. Yes
2. No

**Personal and professional information**

**2. In which country do you work?**

1. Drop-down list of EHRA countries

**3. Which of the following professional titles best describes you?**

1. Nurse
2. Physician associate
3. Cardiology trainee/fellow
4. Cardiology consultant/attending having completed training in the last 10 years
5. Cardiology consultant/attending having completed training greater than 10 years ago
6. Non-Cardiology Physician – Internal Medicine
7. Non-Cardiology Physician – General Practice
8. Other (free text box)

**4. How many electrophysiology procedures have you performed in the last 12 months?**

1. < 50 cases
2. 50 to 150 cases
3. 150 to 400 cases
4. > 400 cases

**Vascular access**

**The questions below relate to electrophysiology procedures only (i.e. cardiac implantable electronic devices are not included).**

**5. Which of the following statements best describes your use of ultrasound guidance for vascular access during electrophysiology procedures?**

1. I always use ultrasound guidance
2. I usually use ultrasound guidance
3. I use ultrasound guidance and landmark techniques with relatively equal frequency
4. I rarely use ultrasound guidance
5. I never use ultrasound guidance

**6. Which of the following statements best describes the availability of an ultrasound machine in your centre when gaining vascular access during electrophysiology procedures?**

1. An ultrasound machine is always available
2. An ultrasound machine is usually available
3. An ultrasound machine is rarely available
4. An ultrasound machine is never available

**7. Have you received formal training on ultrasound-guided vascular access? (for example, have you attended a practical course organised by your institution or externally, or received formal hands-on training including a sign-off process)**

1. Yes
2. No

**8. In your institution, which healthcare professional most often gains vascular access during an electrophysiology procedure?**

1. Doctor – trainee or fellow
2. Doctor – senior cardiologist (consultant or attending)
3. Allied healthcare professional (e.g. physician associate or nurse)
4. The role is shared equally between different healthcare professional groups
5. Other (please specify)

**9. In the last 12 months, which of the following vascular access sites have you used when performing an electrophysiology procedure? Select all that apply. *Please note, epicardial access and surgical access (e.g. vascular cut-down) are not discussed in this question.***

1. Femoral vein (right)
2. Femoral vein (left)
3. Femoral artery
4. Jugular vein
5. Subclavian vein
6. Axillary vein
7. Transhepatic access
8. Other (please specify)

**10. For a routine diagnostic electrophysiology study (i.e. 2-, 3- or 4-wire study), which access site(s) do you most commonly use?**

1. Unilateral femoral vein access only
2. Bilateral femoral vein access
3. Femoral vein access + another access point (e.g. subclavian or jugular vein)
4. Subclavian and/or jugular vein access, without femoral vein access
5. Other (please specify)

**Vascular closure and haemostasis**

In the following section, three different haemostasis techniques are discussed: manual compression, suture-mediated closure (for example, the use of a figure-of-eight suture), and vascular closure devices.

**11. To your knowledge, does your institution have a standardised protocol/policy for vascular closure/haemostasis following electrophysiology procedures?**

1. Yes
2. No
3. I don’t know

**12. Which of the following haemostasis methods do you have clinical experience of using? Select all that apply.**

1. Manual compression
2. Suture-mediated closure
3. Vascular closure device

**(Only to appear if answer to 12 is ‘a’) 13. In your institution, when manual compression is used, who most often performs this?**

1. Doctor – trainee or fellow
2. Doctor – senior cardiologist (consultant or attending)
3. Physician associate
4. Nurse
5. The role is shared equally between different healthcare professional groups
6. Other (please specify)

**(Only to appear if answer to 12 is ‘a’) 14. In an anticoagulated patient undergoing atrial fibrillation catheter ablation, what is, in your experience, the average duration required to achieve haemostasis using manual compression? *Please provide a numerical answer in minutes.***

Answer: free-text box accepting a whole number.

**15. Which of the following suture techniques have you used in clinical practice? Select all that apply.**

1. A figure-of-eight suture secured with a hand-tied knot
2. A figure-of-eight suture secured with a 3-way stopcock (also called ‘3-way tap’)
3. A purse-string suture
4. I have not used any suture techniques in this setting
5. Another suture technique: free-text answer

**16. Which of the following vascular closure devices have you used in clinical practice?**

1. Perclose ProGlide^TM^ (Abbott)
2. VASCADE®(Cardiva)
3. MYNX CONTROL^TM^ (AccessClosure)
4. I have not used any vascular closure devices in this setting
5. Other (please specify)

**17. Across all electrophysiology procedures that you have performed in the last 12 months, in what percentage of cases do you estimate you used a vascular closure device? *Please type the answer as a percentage.***

Answer: slider scale from 0 to 100 %.

**18. To your best guess, what is the average cost of a vascular closure device in euros?**

Answer: slider scale from 0 to 2000 euros.

**19. Which of the following best describes your opinion of vascular closure devices?**

1. Vascular closure devices should be used in all patients undergoing electrophysiology procedures
2. Vascular closure devices should be used in all high-risk electrophysiology procedures (e.g. large-bore access, arterial access), but not in lower-risk procedures (e.g. diagnostic electrophysiology study)
3. Vascular closure devices can be used following electrophysiology procedures, at the discretion of the operator
4. Vascular closure devices should not routinely be used following electrophysiology procedures
5. Vascular closure devices should never be used following electrophysiology procedures

**20. Following electrophysiology procedures, which vascular haemostasis technique do you most commonly use?**

1. Manual compression
2. Suture-mediated closure
3. Vascular closure device
4. Other (please specify)

**21. After achieving vascular haemostasis following an electrophysiology procedure, do you apply a pressure bandage?**

1. Yes, always
2. Yes, sometimes
3. No, never

**(Only to appear if answer to 21 is ‘a’ or ‘b’) 22. When applying a pressure bandage, how long do you usually leave this in place for? Please provide an answer in minutes.**

Answer: slider scale from 0 to 600 minutes.

**Management of periprocedural anticoagulation**

**23. During an electrophysiology procedure requiring transeptal access, at which time point do you first administer therapeutic heparin?**

1. Before venous puncture
2. After venous puncture, but before transeptal puncture
3. After transeptal puncture

**24. When intra-procedural therapeutic dose heparin has been given during an electrophysiology procedure (without arterial access), which of the following statements best describes your use of protamine?**

1. I always give protamine
2. I usually give protamine
3. I sometimes give protamine
4. I rarely give protamine
5. I never give protamine

**(Only to appear if answer to 23 is ‘a’, ‘b’, ‘c’, or ‘d’) 25. When protamine is given, which of the following best describes your practice?**

1. Full-dose protamine is used to fully reverse heparinisation
2. Partial-dose protamine is used to partially reverse heparinisation
3. Either of the above methods can be used

**26. In your routine atrial fibrillation ablation practice, which of the following statements best describes your practice regarding peri-procedural oral anticoagulation?**

1. Oral anticoagulation is always continued uninterrupted
2. Oral anticoagulation is always partially interrupted (for one dose only)
3. Oral anticoagulation is always fully interrupted (i.e. for more than one dose)
4. Oral anticoagulation is sometimes continued and sometimes interrupted

**Bed rest duration prior to ambulation**

**27**. **Does your institution have a standard protocol/policy for bed rest duration following electrophysiology procedures?**

1. Yes
2. No
3. I don’t know

**28. Following a routine right-sided electrophysiology procedure without therapeutic anticoagulation and without intra-procedural complications, what bed rest duration do you usually advise? *Please provide a numerical answer in hours.***

Answer: slider scale from 0 to 24 hours.

**29. Following a routine atrial fibrillation ablation without intra-procedural complications, what bed rest duration do you usually advise? *Please provide a numerical answer in hours.***

Answer: slider scale from 0 to 24 hours.

**30. Following a routine electrophysiology procedure requiring femoral arterial access (e.g. retrograde aortic approach) without intra-procedural complications, what bed rest duration do you usually advise? *Please provide a numerical answer in hours.***

Answer: slider scale from 0 to 24 hours.

**Vascular access site complications**

**31. When consenting a patient prior to an electrophysiology procedure, what do you quote as the risk of vascular complications?**

***Instructions: please ensure you enter a whole number (e. g. 56 or 87) or a decimal number with up to 2 decimal places (e. g. 56.89% or 87.66%)***

Answer: free-text box allowing numerical answer (without answer validation).

**32. Please rate the importance of the following factors for reducing the incidence of access site complications following electrophysiology procedures**

**32a. Mandatory use of ultrasound guidance**

**32b. Mandatory use of vascular closure devices**

**32c. Standardisation of periprocedural anticoagulation protocols**

**32d. Standardisation of bed rest duration**

Answers for each: From 1 [not at all important] to 5 [very important]).

**Supplementary Material – Appendix 2: Bed rest following electrophysiology procedures.** *A*) Existence of standardised institutional policy on bed rest duration. *B*) Recommended bed rest durations (median, interquartile range).


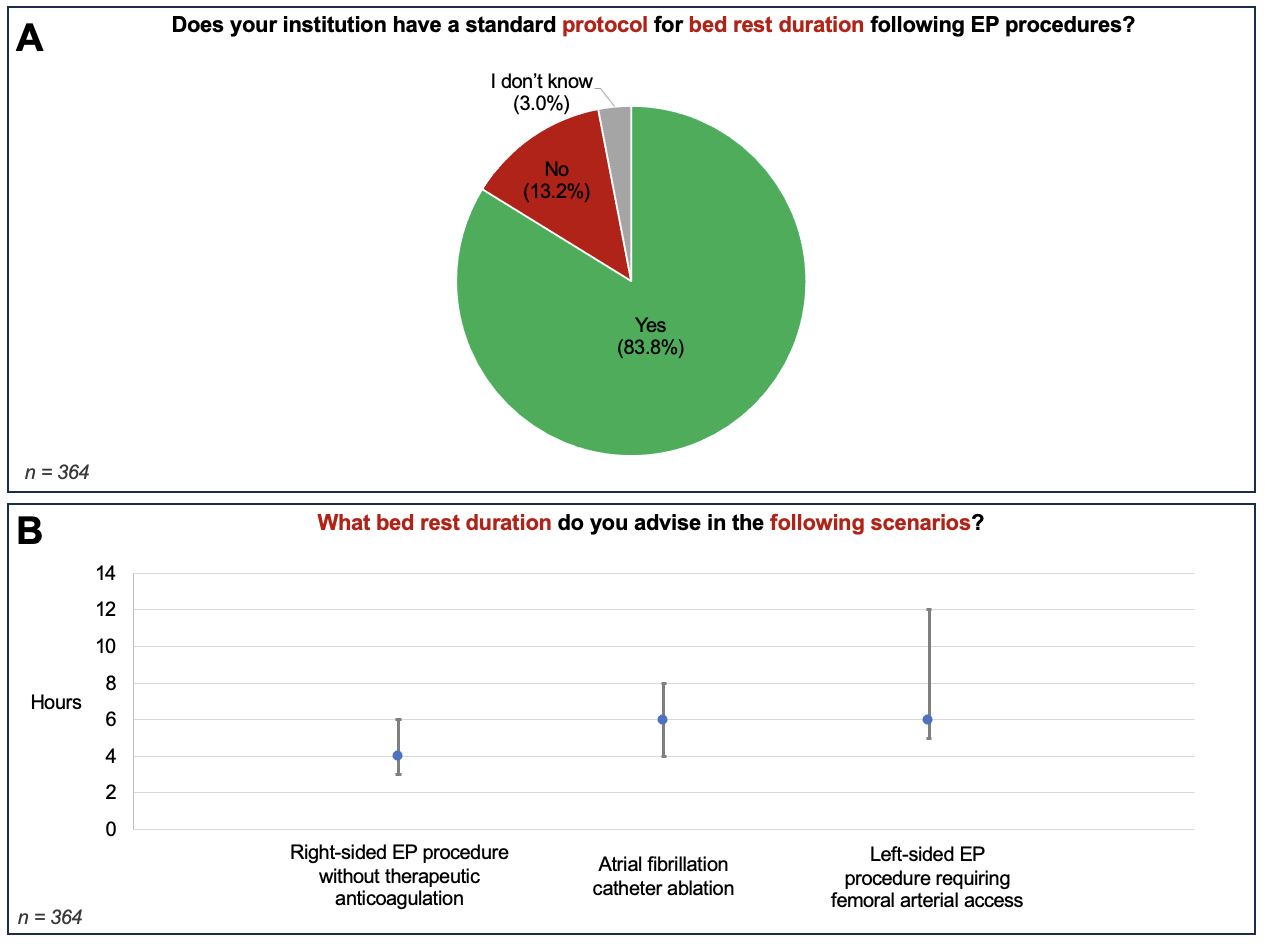

Supplement: euaf117_Supplementary_Data [file euaf117_supplementary_data.docx]
